# Precision wildlife monitoring using unmanned aerial vehicles

**DOI:** 10.1038/srep22574

**Published:** 2016-03-17

**Authors:** Jarrod C. Hodgson, Shane M. Baylis, Rowan Mott, Ashley Herrod, Rohan H. Clarke

**Affiliations:** 1 School of Biological Sciences, Monash University, VIC 3800, Australia

## Abstract

Unmanned aerial vehicles (UAVs) represent a new frontier in environmental research. Their use has the potential to revolutionise the field if they prove capable of improving data quality or the ease with which data are collected beyond traditional methods. We apply UAV technology to wildlife monitoring in tropical and polar environments and demonstrate that UAV-derived counts of colony nesting birds are an order of magnitude more precise than traditional ground counts. The increased count precision afforded by UAVs, along with their ability to survey hard-to-reach populations and places, will likely drive many wildlife monitoring projects that rely on population counts to transition from traditional methods to UAV technology. Careful consideration will be required to ensure the coherence of historic data sets with new UAV-derived data and we propose a method for determining the number of duplicated (concurrent UAV and ground counts) sampling points needed to achieve data compatibility.

The uptake of UAVs in environmental research has been remarkable. In their short history UAVs have been used in applications as diverse as monitoring breeding success of canopy-nesting birds^1^ to surveying elephants^2^. Predictions of ‘big things to come’ for UAV technology are based on the perception that the collection efficiency, cost effectiveness and/or accuracy of data collection using UAVs exceeds that of traditional methods^3^,^4^,^5^,^6^. In wildlife population monitoring applications it is desirable for population counts to be accurate; that is resulting in an estimate that is close to the true population number^7^. In wild populations where the true population size is fundamentally unknown^8^, it is not possible to directly assess the accuracy of any count method. However, it is possible to assess the precision of a count method, defined as the variance between replicated counts by different counters attempting to count the same sample^7^. Regular precise counts facilitate the detection of small magnitude population fluctuations owing to the lower type II error rate in statistical analysis that comes with comparing measures with smaller variance^9^. Reduction in the overall variance exhibited by repeat count data may be obtained by reducing the number of sources of variance in the counting process or by reducing the magnitudes of those variances (Fig. 1). If, as has been predicted, UAV technology results in more precise count estimates than traditional ground-based counting methods then it is likely that many wildlife population monitoring projects will transition from traditional methods to UAV technology for data collection purposes. Transition will require an understanding of how UAV-derived count data compares with that from traditional counting methods so that meaningful comparison can be made between the historical data set and data collected into the future using UAVs. Small UAVs have been used in wildlife monitoring projects to obtain imagery to retrospectively estimate the number of individuals captured in images^10^,^11^. Here, we compare the precision of UAV-derived image counts with counts made concurrently by ground counters for colonies of three seabird taxa – frigatebirds (n = 5), terns (n = 4) and penguins (n = 3) – in tropical and polar environments (Fig. 2). Further, we investigate whether the two count methods return data of comparable magnitude, and propose a method for determining how many times UAV-derived counts and ground counts would need to be carried out in duplicate to ensure compatibility of the two data sets.

## Results

UAV-derived counts had significantly lower variance within colonies than ground counts for all species (frigatebirds: F_1,44_ = 12.294, p = 0.001, Fig. 3a; terns: F_1,44_ = 19.607, p ≪ 0.001, Fig. 3b; penguins: F_1,31_ = 16.075, p < 0.001, Fig. 3c). UAV-derived counts were consistently larger than ground counts for both frigatebirds (Fig. 3a) and penguins (Fig. 3c) in colonies after accounting for variance between colonies (t_40_ = −7.257, p ≪ 0.001; t_29_ = −0.377, p ≪ 0.001, respectively), however, the magnitude of the difference between ground and UAV-derived counts varied substantially between penguin colonies (Fig. 3c). There was no significant difference between ground and UAV-derived counts of terns in colonies (t_41_ = 0.694, p = 0.492; Fig. 3b) although median ground counts were lower than median UAV-derived counts for all colonies.

Simulated data matching our observed ratio of ground counts to UAV-derived counts (G:U) and variances of count magnitudes for frigatebirds and terns indicated approximately 15 duplicate counts are required before estimation error below +/− 10% of the true G:U is attained (Fig. 4, Table S1). For monitoring projects aiming to maintain error at or below +/− 5% during transition from ground counts to UAV-derived counts, 50 duplicate counts were required in the frigatebird simulation whereas approximately 80 duplicate counts were required in the tern simulation. In interpreting these simulations, we stress that they are approximations only, informed by a single field season and are expected to vary by species and with environmental conditions. However, if seasonal or colony-specific variations in G:U are small, then these models will give a good approximation of the number of samples necessary to make interpretations between ground and UAV-derived counts at a desired degree of accuracy.

## Discussion

**Figure 1:**
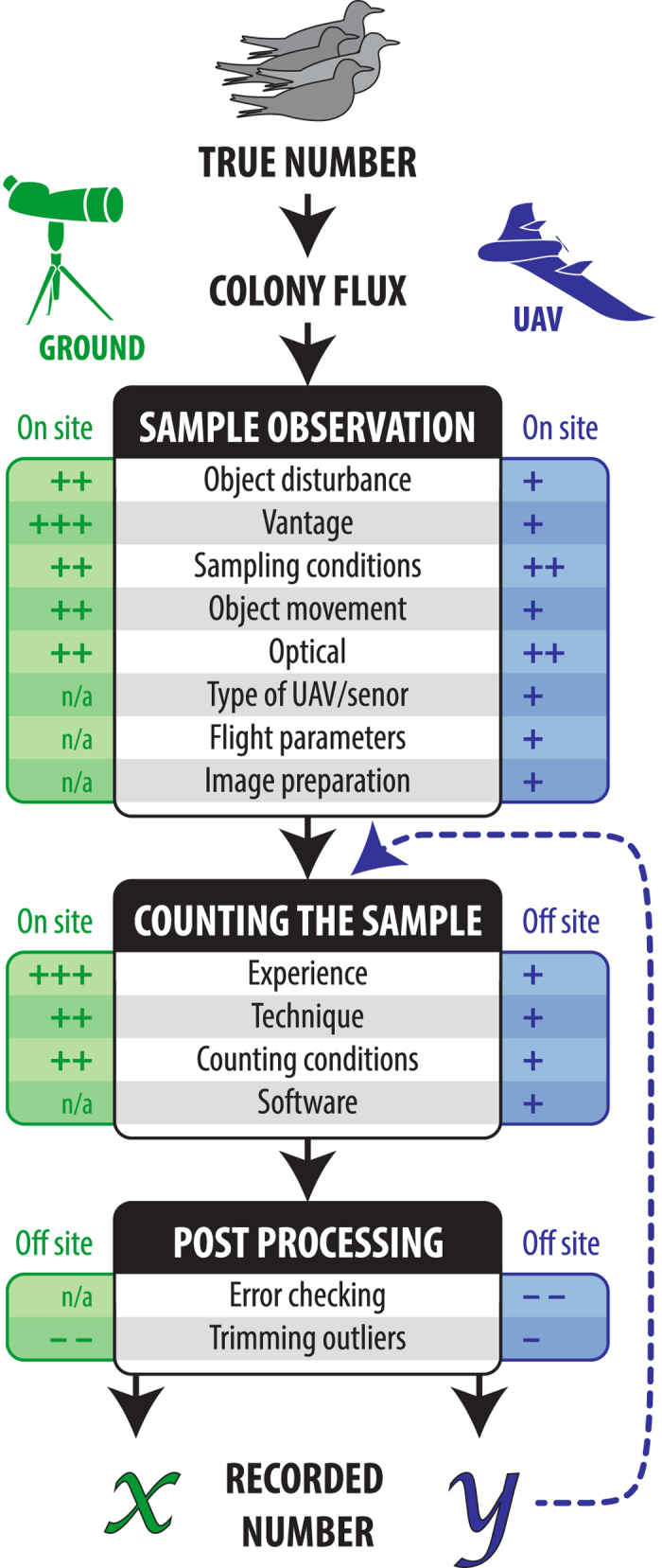
Potential sources of variance when estimating the number of subjects in a faunal aggregation using a traditional ground (green) or UAV (blue) counting technique. The estimated magnitude of variance for each element for each count type either increases (+) or reduces (−) variance in estimates at a minor (+), moderate (++) or major (+++) scale. We categorised elements into three main stages which occur on or off site: (a) observing the sample, (b) counting the sample and (c) post processing. The hashed blue arrow indicates the ability for UAV-derived counts to be repeated using the existing sample. Figure created by the authors.

**Figure 3:**
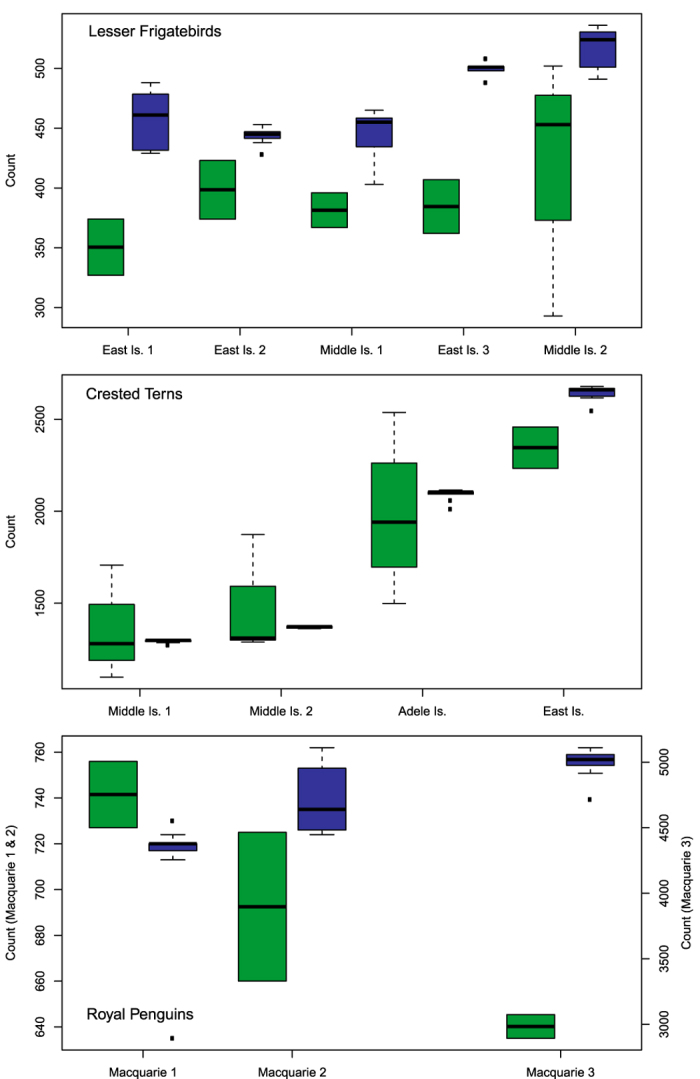
Colony size estimates made by experienced ground counters using a traditional technique (green; n = 2–4) and those made by counting digital imagery captured by a small UAV (blue; n = 7–9) which was flown concurrent to ground surveys. Colonies of varying size were sampled on Australian islands: breeding Lesser Frigatebirds *Fregata ariel* (a) and Crested Terns *Thalasseus bergii* (b) at the tropical Ashmore Reef Commonwealth Marine Reserve as well as on the nearby Adele Island in April 2014 and moulting Royal Penguins *Eudyptes schlegeli* (c) on the subantarctic Macquarie Island in April 2015.

**Figure 4:**
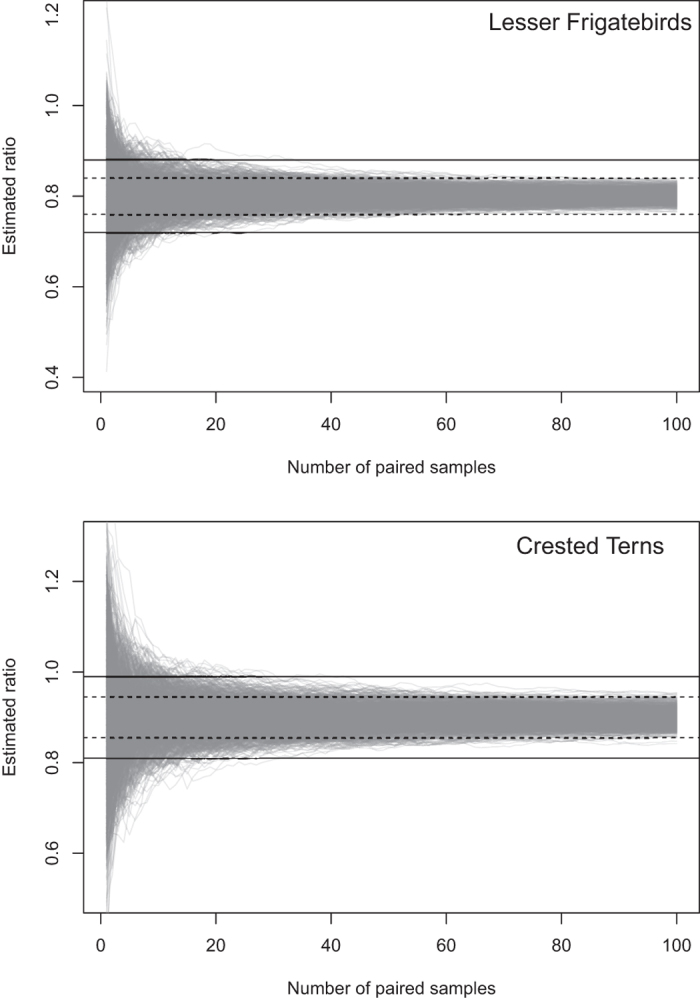
Simulated accuracies of ground count:UAV-derived count ratios under increasing numbers of paired samples. For Lesser Frigatebirds *Fregata ariel* and Crested Terns *Thalasseus bergii*, the ground and UAV-derived count means and variances are set at the values provided in Supplementary Table 1. Each grey line represents the cumulative estimate of the G:U ratio over an increasing number of paired samples. Dashed black lines represent +/−5% of the true ratio, and solid black lines represent +/− 10% of the true ratio.

We demonstrate that UAV-derived estimates of colony size result in smaller cumulative variance compared to conventional ground-based approaches (Fig. 1). While enhanced precision does not guarantee greater estimate accuracy, it does increase the power of our statistical ability to detect population trends^9^. We expect this same benefit to extend to other animal groups and geographic contexts. We also find that UAV-derived counts are consistently similar to or significantly larger than ground counts. The nadir (downward-facing) perspective of UAV imagery reduces the likelihood of missed counts due to topography and birds obscuring the counters’ line of sight. Additionally, still imagery from UAVs presents the option of separating the count area into manageable subsets and completing counts in multiple sittings.

However, the transition from traditional to new UAV-based monitoring methods requires careful consideration, particularly in terms of maintaining the relevance of historical data that have been collected at substantial time and financial cost^12^,^13^. Our simulations indicated that the true ratio of the magnitude of ground count to UAV-derived count (G:U) can be estimated to an accuracy of +/− 10% after completing approximately 15 duplicate counts for frigatebirds and terns. Substantially more counts are required to reduce this error margin to within +/− 5%. However, this simulation was not carried out for penguins as the ratio of ground to UAV-derived counts was not consistent between colonies. Although our data indicate that ground surveys typically resulted in smaller estimates than UAV-derived counts, other studies indicate the converse is possible^14^,^15^. Regardless of the direction of the relationship, if a consistent G:U and a corresponding error metric can be established, as with our terns and frigatebirds, then data continuity is ensured.

This research demonstrates that the precision of population estimates of seabirds in both tropical and polar environments can be improved using UAV technology compared to ground counts. We also present an analytical model to facilitate the transition of monitoring programs to this more precise technique. Our findings suggest that population estimates using UAVs could be a powerful new tool in the ecologist’s tool kit for precision wildlife monitoring.

## Methods

### Study sites and species

Five Lesser Frigatebird *Fregata ariel* (frigatebird) and two Crested Tern *Thalasseus bergii* (tern) colonies were sampled at two tropical islands at Ashmore Reef Commonwealth Marine Reserve, Australian (12°16′S 123°2′E), and one tern colony at nearby Adele Island, Western Australia (tropical; 15°31′S 123°9′E). Frigatebirds nest on the ground among herbaceous vegetation at these locations while terns nest in a simple sand scrape in colonies situated on un-vegetated, sandy shorelines.

Three colonies of moulting Royal Penguins *Eudyptes schlegeli* (penguins) were sampled at subantarctic Macquarie Island, Australia (54°30′S 158°56′E) in April 2015. Sampled colonies were situated on a bare soil substrate. Individuals were at various stages of moult.

Research was conducted in accordance with wildlife research (Parks Australia: 066 ARRR-110217-01; Department of Parks and Wildlife: CE004403, Tasmania Parks and Wildlife Service: RAA 2925) and animal ethics (Monash University Animal Ethics Committee: BSCI/2010/08, BSCI/2012/08 and BSCI/2014/09) permits. Experimental protocols were approved by the Monash University Animal Ethics Committee (see permits details above).

### Ground counts

Ground counts were made by experienced seabird counters using standard techniques^16^. Counters used tripod-mounted spotting scopes, binoculars or the naked eye as appropriate. Hand-held two-bank tally counters were used to assist counting. Observation viewpoints were at the same altitude as the sample except one colony (Macquarie 3) which had a 5 m altitude vantage. Counting commenced immediately post UAV recovery. Two to four ground counters each made a single count of the number of individuals within a defined colony. The duration of each count varied (10–30 minutes) depending on colony size. Although there may have been some flux of individual birds over the short period between obtaining aerial imagery and completing ground counts (15–45 minutes), we assumed that the colony numbers were constant given individuals within the sample were either tending a nest (tropical colonies) or moulting (polar colonies).

### UAV flight parameters

Tropical colonies were photographed using a small, off-the-shelf octocopter (X8, 3D Robotics – approximate cost: US$1,500). Missions were flown autonomously along a pre-programmed flight path (Mission Planner, planner.ardupilot.com) at a speed of 2–3 m/s and altitude of 75 m above surface level (asl), with launch and recovery greater than 100 m from sampled colonies. Two passes were made over the target colony before returning to the launch site. Total flight time for each colony was 4–12 minutes.

Polar colonies were sampled using a custom flying wing conservation drone (FX79 airframe; in association with HornbillSurveys.com – approximate cost: US$3,000) flown at approximately 13 m/s at 120 m asl. Ground speed was variable relative to weather conditions and pre-programmed, autonomous missions (as per tropical colonies but with manual launch and recovery) were executed. Total flight for each colony was approximately 10–20 minutes.

For all colonies, imagery was captured using a mirrorless digital SLR camera (EOS M, Canon – resolution: 5184 × 3456 px; sensor: CMOS; sensor size: 22.3 × 14.9 mm; approximate cost: US$450) with 40 mm lens (EF, Canon – approximate cost: US$150) and UV filter (Hoya). The manufacturer’s firmware was replaced (MagicLantern, magiclantern.fm) to control the camera’s intervalometer and photograph successively in flight. Photographs were taken at 2–3 second intervals with 1/400–1/1600 second shutter speed, and shot at the highest possible quality in jpeg format. The camera was mounted facing downward and vibration blur was mitigated using a commercial vibration dampening plate for the octocopter and iSPONGE^3^ for the flying wing.

Colonies were observed by counters leading up to and during the UAV overflight, as any group startle response which resulted in birds taking flight would obviously affect recorded counts. No group startle response was observed during UAV counts.

### Counting aerial images

Unaltered images of a colony were merged in Adobe Photoshop (CS6) to form a composite of the entire colony. Geometric corrections were not considered important and may have distorted the shape of birds in the image. Counts were conducted on a desktop computer. As not all counters were bird specialists, all counters were shown high resolution close up photographs of each species to ensure they could effectively differentiate them from other birds. A grid was overlaid on all files and systematic counts, gridcell-by-gridcell, were then made (left to right, top to bottom) in a laboratory environment using Photoshop’s count tool. Flying birds were ignored. Counters were encouraged to zoom in to each cell as they progressed and, upon completion, review their count at different levels of zoom until they were satisfied they had counted all individuals. Seven to nine people made blind counts of each colony. The time taken to complete each colony count varied (15–90 minutes). Some ground counters were also UAV counters, although not every ground counter counted every drone image, or vice-versa.

### Statistical methods

All analyses were carried out in R 3.1.2^17^. Ground and UAV-derived counts were considered linked – i.e., for each flight, ground counters counted the colony immediately after UAV recovery, so analyses treated these as paired samples.

The equality of variances between ground and UAV-derived counts was assessed using a Levene’s test to test for a difference in variance of the residuals from the models for count size between ground and UAV-derived counts. Each species was tested separately.

In order to determine whether ground counts and UAV-derived counts were equal in magnitude, we fitted a mixed-effects Poisson model^18^ using the glmmPQL function in the MASS package^19^, with counts modelled as a function of count type (ground or UAV-derived count). Colony identity was included in the model as a random effect. Because an early model showed a significant interaction effect between species and count type on count, each species was modelled separately.

We carried out a simple simulation model to determine how many duplicate counts using both count techniques should be undertaken so that trends are interpretable between the two data collection methods. UAV-derived counts had a higher median than ground counts for all but one of the colonies. This simulation was not carried out for penguins as the ratio of ground to UAV-derived counts was not consistent between colonies. Ground counts of frigatebirds recorded approximately 80% of the individuals in a matched UAV-derived count, whereas ground counts of terns recorded approximately 90% of the individuals in a matched UAV-derived count. As our counts were large (>100 s), we employed a Normal approximation for our simulations. The model simulated paired samples of ground and UAV-derived counts with the underlying ratio of ground counts to UAV-derived counts, and the variance of ground counts and UAV-derived counts, set to reflect the ratios and variances in our data. For each set of samples, we plotted the cumulative ratio of ground counts to UAV-derived counts across increasing numbers of paired samples. For each of 1000 iterations, we simulated 100 paired samples to estimate how many paired samples were necessary in order for the cumulative ratio to reflect the underlying ratio within a given tolerance range of the true ratio with a given probability.

## Additional Information

**How to cite this article**: Hodgson, J. C. *et al*. Precision wildlife monitoring using unmanned aerial vehicles. *Sci. Rep*. **6**, 22574; doi: 10.1038/srep22574 (2016).

## Supplementary Material

Supplementary Information

## Figures and Tables

**Figure 2:**
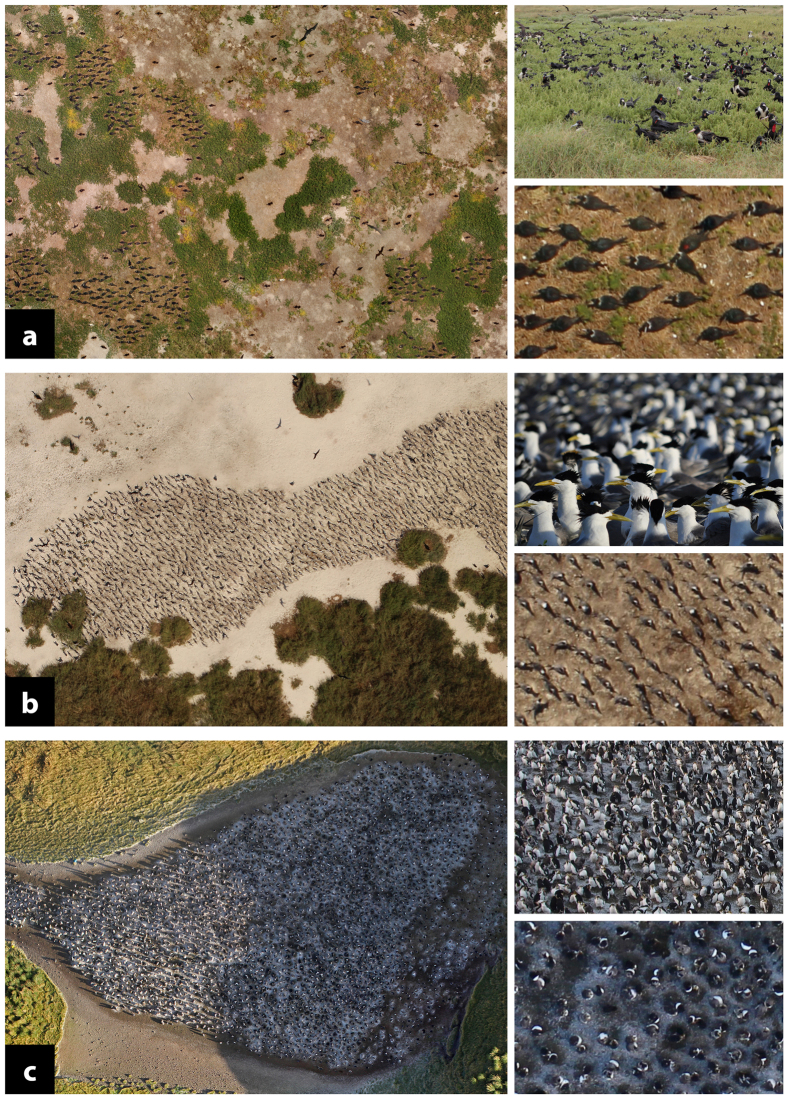
Imagery of sampled colonies of seabirds on remote Australian islands: breeding Lesser Frigatebirds *Fregata ariel* ((**a**) April 2014), breeding Crested Tern *Thalasseus bergii* ((**b**) April 2014), and moulting Royal Penguins *Eudyptes schlegeli* ((**c**) April 2015). The main image for each species is comprised of several photographs captured by a UAV and merged to make estimates of the number of individuals within the colony. The lower inset of each panel is a magnified view of the main image. Upper insets illustrate the vantage of a ground counter. Images are not to scale and are by Jarrod Hodgson.
