# Supplementary material for: Precision wildlife monitoring using unmanned aerial vehicles
Source: Sci Rep. 2016 Mar 17;6:22574. doi: 10.1038/srep22574 (PMC4795075; doi:10.1038/srep22574)
Supplement: Supplementary Information [file srep22574-s1.pdf]

Title: Precision wildlife monitoring using unmanned aerial vehicles

Authors: Jarrod C. Hodgson[1, 2] *, Shane M. Baylis[1], Rowan Mott[1], Ashley Herrod[1] & Rohan H. Clarke[1]

Affiliations:

[1] School of Biological Sciences, Monash University, VIC 3800, Australia.

[2] Present affiliation: School of Biological Sciences, The University of Adelaide, SA 5005, Australia.

*Correspondence to: jarrod.hodgson@adelaide.edu.au

Supplementary Materials:

Supplementary Table 1: Estimates of stable ratios between ground and UAV-derived counts for long-term monitoring projects. We consider that UAV-derived counts may consistently over or under estimate colony sizes relative to matched ground counts. In that case, establishing the long-term ratio of ground counts to matched UAV-derived counts will be of interest to researchers conducting long-term monitoring studies, to allow comparison of estimates made by the two methods. Here, we present the number of paired samples necessary to estimate the ratio of ground to UAV-derived counts to a desired level of accuracy at a desired level of confidence. The underlying ratio of ground to UAV-derived counts are taken from our counts for Lesser Frigatebirds *Fregata ariel* and Crested Terns *Thalasseus bergii*, which appeared to show a consistent ratio across all colonies. Our full method for estimating the required number of samples, which may be updated to provide estimates for users' datasets, is presented as Electronic Supplementary Materials.

**Ground mean (SD)**	**UAV mean (SD)**	**Tolerance level (%)**	**Confidence level**	**Number of samples**
Lesser Frigatebirds				
400 (55)	500 (20)	10	0.95	7
400 (55)	500 (20)	10	0.99	12
400 (55)	500 (20)	5	0.95	33
400 (55)	500 (20)	5	0.99	52
Crested Terns				
1800 (320)	2000 (45)	10	0.95	13
1800 (320)	2000 (45)	10	0.99	21
1800 (320)	2000 (45)	5	0.95	49
1800 (320)	2000 (45)	5	0.99	89

Extended methods – analysis code

The following script simulates paired samples of a colony or species using both UAV counting and ground counting techniques. It outputs plots and data summaries useful for determining the number of paired samples that may be required in order to infer the ratio of UAV counts to ground-counts, in species where UAV counts consistently under- or over-estimate population sizes, relative to ground counts. The final plot shows the proportion of trials where the estimated ratio is within 'tolerance' of the true, underlying UAV:Ground count ratio at each progressive number of paired samples. Lines are at 95% of trials within 'tolerance' # and 99% of trials within 'tolerance'.

```
## This code was written in R Version 3.2.1 under Emacs and ESS.
```

```
## The following script is from Hodgson, J. C., Baylis, S. M., Mott, R.,
 # Herrod, A. & Clarke, R. H.  (2016) "Precision wildlife monitoring
 # using unmanned aerial vehicles", in Scientific Reports.
 # It is shared under a Creative Commons Attribution 4.0
 # International license.

dronemean <- 2000
dronesd <- 45
groundmean <- 1800
groundsd <- 320  ## drone and ground means and sd's of counts
indexLength <- 100  # the maximum number of paired samples in the simulation
repNum <- 1000      # the number of simulations to run
trueRatio <- groundmean/dronemean
tolerance <- 0.05  # the desired level of accuracy
tolerance2 <- 0.1 # the outer level of accuracy - plotted only
upperPoint <- trueRatio+(trueRatio*tolerance) # upper limit of tolerance
upperUpperPoint <- trueRatio+(trueRatio*tolerance2)
lowerPoint <- trueRatio-(trueRatio*tolerance) # lower limit of tolerance
lowerLowerPoint <- trueRatio-(trueRatio*tolerance2)

index <- c(1:indexLength )
droneNumbers <- rnorm(indexLength, dronemean, dronesd)
groundNumbers <- rnorm(indexLength, groundmean, groundsd)

ratios <- groundNumbers/droneNumbers

cumrat <- rep(0,indexLength)   ## the 'cumulative ratio' of ground to
                               # drone counts for all samples
                               # collected so far.
for(i in 1:length(cumrat)) {
```

```
  cumrat[i] <- mean(ratios[1:i])
}


plot(cumrat~index,type="l",
     ylim=c(0.5,1.3),col= grey(0.5, alpha=0.17),
     xlab="Number of paired samples", ylab="Estimated ratio")
text(85,1.3,"Crested Terns",cex=1.5)  ## Name your species here


allFrames <- data.frame(repNo=c(0),index=c(0),cumrat=c(0.00010))


for(j in 1:repNum){
  droneNumbers <- rnorm(indexLength, dronemean, dronesd)
  groundNumbers <- rnorm(indexLength, groundmean, groundsd)
                  # current ratio is ground = 0.8(drone)
  ratios <- groundNumbers/droneNumbers


  cumrat <- rep(0,indexLength)
  for(i in 1:length(cumrat)) {
    cumrat[i] <- mean(ratios[1:i])
  }
  lines(cumrat~index,col=grey(0.5,alpha=0.17))


  repNo <- rep(j,indexLength)
  currentFrame <- data.frame(repNo,index,cumrat)
  allFrames <- rbind(allFrames,currentFrame)
}


abline(upperPoint,0,col="black",lty=2)
abline(lowerPoint,0,col="black",lty=2)
abline(upperUpperPoint,0,col="black",lty=1)
```

```
abline(lowerLowerPoint,0,col="black",lty=1)
## dashed lines indicate true ratio +/- 10% of that ratio


allFrames <- allFrames[2:nrow(allFrames),]
## allFrames is a three-vector dataframe, with variables repNo,
 # index, and cumrat. repNo is a numeric label for which 'line'
 # the datapoint is from, index is the order within that line,
 # and cumrat is the cumulative ratio at that point on that line.


proportionInRange <- c(rep(0,indexLength))
for(k in 1:indexLength){
    indexPoint <- subset(allFrames,index==k)
    proportionUnderMax <- subset(indexPoint,cumrat<upperPoint)
    proportionOverMin <- subset(proportionUnderMax,cumrat>lowerPoint)
    proportionInRange[k] <- nrow(proportionOverMin)/nrow(indexPoint)
}
plot(proportionInRange, xlab="Number of paired samples",
     ylab="Proportion of estimates within the desired accuracy range")
     ## this plot shows the proportion in range, over count-repeats
abline(0.99,0,col="black",lty=2)
abline(0.95,0,col="black",lty=2)
```
